# Personal and Social Resources at Work: Reciprocal Relations Between Crafting for Social Job Resources, Social Support at Work and Psychological Capital

**DOI:** 10.3389/fpsyg.2019.02632

**Published:** 2019-11-22

**Authors:** Philipp Kerksieck, Georg F. Bauer, Rebecca Brauchli

**Affiliations:** Institute of Epidemiology, Biostatistics and Prevention, Public and Organizational Health, University of Zurich, Zurich, Switzerland

**Keywords:** psychological capital, social support, job crafting, job demands – resources theory, enabling hypothesis

## Abstract

The availability and development of social and personal resources are substantial components of a positive work experience. This study aims to inquire the reciprocal relations between the personal resource of psychological capital (PsyCap; hope, self-efficacy, resilience, and optimism) and the social job resource of social support, as proposed in the job demands-resources theory. There, job crafting is defined as a catalysator to the interplay of social support and PsyCap and is therefore added to this study. Moreover, we test the enabling hypothesis of social support in the context of work. We contribute to the field, as this research (a) examines propositions of a core theory, (b) adds and extends relevant hypotheses from health psychology into occupational psychology, and (c) aims to replicate findings. To capture the dynamic nature of the selected, relevant relationships of the job demands-resources theory, we used a three-wave, 3-month panel design to study 995 employees who were working in a broad range of economic sectors and occupations. Structural equation modeling was used to test hypotheses. Results showed, that social support at work positively influenced the development of PsyCap, supporting and extending the enabling hypothesis of self-efficacy. Counterintuitively, PsyCap and crafting for social job resources were negatively related, indicating (a) that the reliance on personal resources might reduce the necessity to generate social resources, and (b) that crafting is a strategy that consumes personal resources. Previously observed gain cycles were not replicable.

## Introduction

Today, the so-called digital, or fourth industrial revolution, is ever-present. This development is noticeably changing the face of work in a significant haste, offering new options for employees and organizations, and at the same time poses challenges to both (Neufeind et al., 2018). Such a development implies a considerable demand for social and personal resources, to adapt to and to actively craft changing work situations. Thus, both, social resources, such as peer and supervisor support, as well as personal resources, as self-efficacy, optimism, hope and resilience, are becoming more and more important. At the same time, new technologies facilitate the development of new working modes, including crafting job demands-resources at work, in accordance with own needs and standards. To our best knowledge, this is the first study that combines crafting with individual and social resources in the field of occupational health psychology, adding hypotheses regarding self-efficacy to the study framework.

These entities are captured and theoretically framed in one of the most established theories in the field of occupational health psychology, that is the job demands-resources (JD-R) theory (Demerouti et al., 2001; Bakker and Demerouti, 2017)^1^. The JD-R theory considers the interplay of job demands and resources as relevant for overall job performance (Bakker and Demerouti, 2017). This theory is the overarching framework for our study and defines two entities:

*Job demands are those physical, social, or organizational aspects of the job that require sustained physical and/or psychological effort and are therefore associated with physiological and/or psychological costs. *Job resources* are those physical, social, or organizational aspects of the job that (a) are functional in achieving work-related goals, (b) reduce job demands and the associated physiological and psychological costs, and (c) stimulate personal growth and development (Xanthopoulou et al., 2007, p. 122)*.

For the development of our hypotheses, we highlight two aspects of the JD-R theory (Bakker and Demerouti, 2017, p. 275): Aspect 1 holds the assumption that personal resources could have similar functions as job resources. In addition, and for this study of particular interest, this aspect holds that both personal and job resources interact in a positive, self-reinforcing way with each other. This interaction is conceptually preceded by aspect 2, which states that job crafting leads to higher levels of both, personal and job resources.

Designing a job according to one’s need, in other words, “crafting the job” (Wrzesniewski and Dutton, 2001), is a popular concept in the field of occupational health psychology. Crafting is the subject of a large body of literature. According to Tims et al. (2012) proactive job crafting aims for four key elements of the JD-R theory: (1) increasing structural job resources; (2) increasing social job resources; (3) increasing challenging job demands; (4) decreasing hindering job demands. Empirically, Tims et al. (2013) showed in a three-wave longitudinal study that job crafting predicted substantial changes in job demands and resources in general. Specifically, crafting for *social job resources* increased well-being (i.e., work engagement and job satisfaction) and decreased burnout.

As social job crafting seems to be crucial, recent studies have emphasized the need for more research on this social aspect of job crafting (Rofcanin et al., 2018; Zhang and Parker, 2018). Thus, in this study, we explicitly focus on crafting for social job resources. Regarding social job resources, we focus on social support at work as a highly important job resource. As a key aspect of the basic human need for relatedness (Ryan and Deci, 2000), social support at work is relevant for job satisfaction and job tenure (Harris et al., 2007), and the reduction of work to family conflicts (Kossek et al., 2011). It has also been identified as a relevant buffer in overall work stress and strain (for a review see Viswesvaran et al., 1999), work-family conflict (Kossek et al., 2011), and turn-over intention (Nohe and Sonntag, 2014).

Because job crafting is a strategy used to establish a fit between the person and the environment, not only the social context (in the context of this study, the degree of social support at work) but also the person and his or her specific characteristics are of great importance, which is reflected in the JD-R theory (Bakker and Demerouti, 2017). According to this theory, job crafting has a positive effect on both job resources and personal resources. Therefore, in this study, we also focus on personal resources that are operationalized by the well-established concept of PsyCap, which is conceptualized as a state-like, higher-order factor that contains four subdimensions: hope, self-efficacy, resilience, and optimism (Luthans and Youssef, 2004). Regarding the relevance of PsyCap for the field of occupational health psychology, Avey et al. (2010) found that PsyCap was positively related to extra role organizational citizenship behaviors and negatively related to organizational cynicism, intention to quit, and counterproductive workplace behaviors. Additionally, these authors were able to predict self-evaluation, person-to-organization fit, and person-to-job fit by applying PsyCap. To study PsyCap, we integrate them into two hypotheses, that stem from self-efficacy research. To our knowledge, neither the enabling nor the cultivation hypothesis were verified with PsyCap, additionally, not in the work context. Beyond that, this study contributes to the field as it offers a broad picture by integrating relevant variables into a joint model.

### Aim and Hypotheses

This study aims to systematically assess the interactions among three highly important concepts as predicted by the JD-R theory: Crafting for social job resources, social support at work, and PsyCap. Previous research has provided fragmented evidence for unidirectional as well as reciprocal interactions among these concepts. In the following, we review this literature to develop hypotheses regarding unidirectional relationships of all three variables. We then introduce reciprocal hypotheses.

### Social Support at Work Leads to PsyCap and Vice Versa

Job resources, including social support at work, resulted in a higher dedication to work and a stronger organizational commitment (Bakker et al., 2003). Moreover, social support is an important source of extrinsic motivation in the workplace (Bakker, 2008). The *enabling hypothesis* (Benight and Bandura, 2004) states that social support enables self-efficacy by the positive experience of success and mastery. Through this psychosocial mechanism, one may assume, that social support at work fosters PsyCap in general. As an experience of success and mastery, this mechanism will also positively influence other subdimensions, such as hope, resiliency, and optimism. In summary, “events that are currently occurring in an employee’s social environment continuously shape his/her confidence, hope, optimism, and resiliency” (Luthans and Youssef, 2004, p. 157). Therefore, we hypothesize the following:

Hypothesis 1a: Social support at work at T1 has a positive, cross-lagged effect on PsyCap at T2 and correspondingly from T2 to T3.

The *cultivation hypothesis* (Schwarzer and Knoll, 2007) states that self-efficacy enables social support. That is, because self-efficacious persons expect positive outcomes from their interactions with other persons, they will actively seek social interactions (Alessandri et al., 2009). Additionally, higher levels of self-efficacy result in positive perceptions of the received social support at work (Borgogni et al., 2011). In summary, individuals with high self-efficacy are more prone to interact in everyday work settings, which is in accordance with other PsyCap subdimensions. So far, there is no evidence above and beyond self-efficacy regarding this. Because of this, we will extend the cultivation hypothesis, which is so far specified on self-efficacy, and integrate the entire PsyCap complex into our research hypothesis. This is done, because positive psychological constructs, and in addition to self-efficacy, also optimism, resilience and hope may enable social support. We therefore hypothesize:

Hypothesis 1b: PsyCap at T1 has a positive, cross-lagged effect on social support at work at T2 and correspondingly from T2 to T3.

### Does Social Support Lead to Crafting for Social Job Resources and Vice Versa?

Most workplaces are shaped by social interactions (Grant and Parker, 2009). Job crafting depends on the quality of the social relations and interactions at work (Rofcanin et al., 2018), also because colleagues transmit their job crafting styles among each other (Demerouti and Peeters, 2018). Individual job crafting contributes to the proactivity of colleagues and team members and to their own job crafting behavior; it therefore seems to be a socially reciprocal concept that relies on social interaction (Bakker et al., 2015). It could be imagined that a stimulating and positive framework of social support at work would be a job resource that fostered individuals’ opportunities at their workplace. In detail, crafting for social job resources refers to the resources social support, supervisory coaching, and feedback, as proposed by Tims et al. (2012). Hence, crafting might be executed according to agreements with colleagues and supervisors. Work–home interactions and others then could be crafted according to the individual’s needs and standards. Therefore, job crafting could be supported and shaped by social support at work and depends on the quality of the social interactions. Therefore, we hypothesize the following:

Hypothesis 2a: Social support at work at T1 has a positive, cross-lagged effect on crafting for social job resources at T2 and correspondingly from T2 to T3.

Can crafting for social job resources also contribute to social support at work? It has been documented that job crafting based on its social dimension can result in a substantial improvement in job resources on a general level (Tims et al., 2013). Thompson (2005) applied a social capital perspective to explain the positive interaction between the proactive personality and job performance. The social capital concept of Nan Lin (2002), to which Thompson referred, contains three elements: “resources embedded in a social structure; accessibility to these social resources by individuals; and use or mobilization of them by individuals *engaged in purposive action*” [Lin et al., 2001, p. 58 (emphasis added)]. These elements fit the association between crafting for social job resources and social support at work because they involve the action orientation of social capital. Crafting for social job resources reflects the proactive mobilization of social capital and therefore social support in workplace settings. Thus, we hypothesize that crafting for social job resources had a relevant effect on the extent of received social support:

Hypotheses 2b: Crafting for social job resources at T1 has a positive, cross-lagged effect on social support at work at T2 and correspondingly from T2 to T3.

### PsyCap Leads to Crafting for Social Job Resources and Vice Versa

In the present study, we are interested in the extent to which a person’s PsyCap leads to a proactive action to specifically craft his or her working conditions. Bakker et al. (2012) showed that persons with proactive personalities, that can be seen as a personal resource and an individual level prerequisite to crafting, are more likely to change job-related circumstances in a positive way, and the same research group outlined that self-efficacy as a subdimension of PsyCap, is related to general job crafting (Tims et al., 2014).

Previous research has indicated, that individual factors, such as the Big Five personality traits, are predictors of job crafting. For example, increasing social job resources was significantly correlated with Agreeableness, Extraversion, and Openness to experience (Bell and Njoli, 2017). Consequently, a person with high PsyCap might craft a job more likely. This is, as optimism and hope are linked to motivation, and individuals with high optimism are more likely to engage in proactive behaviors or goal engagement, and they are better at balancing effort expenditures (for an overview see Carver and Scheier, 2014). A subdimension of PsyCap, self-efficacy is related to general job crafting (Tims et al., 2014), and it may specifically foster crafting for social job resources. Overall, it seems plausible that PsyCap might motivate an individual to behave proactively in general (Luthans et al., 2007b). Therefore, we hypothesize:

Hypotheses 3a: PsyCap at T1 has a positive, cross-lagged effect on crafting for social job resources at T2 and correspondingly from T2 to T3.

The dimensions of PsyCap have been characterized as state-like and open to development (Luthans et al., 2007), and they have been shown to alter and change significantly (Luthans et al., 2010). Moreover, because of its plasticity, training to foster PsyCap has been emphasized (Luthans et al., 2012). In a quasi-experimental field study, Van den Heuvel et al. (2015) found that an intervention led to more affective well-being and self-efficacy through job crafting. Previous research also provided evidence for the contribution of job crafting to all PsyCap subdimensions (Vogt et al., 2016). Thus, the context in which one works and the opportunities to craft the job context, might have a substantial impact on the subdimensions of PsyCap. For example, employees who successfully adapted their working circumstances by crafting, may experience a positive result of their efforts in personal resources, as formulated in aspect 2 of the JD-R theory (Bakker and Demerouti, 2017). Consequently, PsyCap subdimensions such as hope, resilience and optimism will increase and accumulate as a result of this experience. Hence, the impact of job crafting on PsyCap could be interpreted as the generalization of self-determined workplace experiences to the accumulation of personal resources. Therefore, we hypothesize:

Hypotheses 3b: Crafting for social job resources at T1 has a positive, cross-lagged effect on PsyCap at T2 and correspondingly from T2 to T3.

### Reciprocal Interactions or Gain Cycles Between Psychological Capital, Social Support at Work, and Crafting for Social Job Resources

So far, we outlined evidence that positive interactions between 2 of the studied concepts are plausible. In the following, we therefore investigate the reasonable question whether all 3 concepts interact in a self-reinforcing positive gain cycle or even spiral which each other.

Several studies in the field of occupational health psychology have reported reciprocal relationships among job resources, personal resources, and health and well-being. For instance, a longitudinal study among school teachers found that personal resources (efficacy beliefs) and organizational resources (social support orientation, innovation orientation, rules orientation, and goals orientation) fostered flow experiences, which predicted future resources (Salanova et al., 2006). Hakanen et al. (2008) reported positive reciprocal associations among job resources, work engagement, and personal initiative. Similarly, Salanova et al. (2011) showed the reciprocal influence of the personal resources of efficacy and work engagement. In another study, interactions among PsyCap, job performance, and work engagement during a 1-year span indicted a gain cycle of respective variables (Alessandri et al., 2018).

In combination, aspect 1 and 2 of the JD-R theory form a starting point for a gain cycle as job resources and personal resources are hypothesized mutually re-enforcing and as job crafting is hypothesized to lead to higher levels of both job and personal resources. According to the conservation resources theory (COR; Hobfoll, 1989, 2001), individuals conserve existing resources and accumulate new resources to buffer the effects of stress. The theory was successfully adapted to the field of occupational health psychology in order to explain gain cycles (Llorens et al., 2007; Harju et al., 2016). Therefore, we expect to find gain cycles of social support, PsyCap, and crafting for social job resources because (a) resources were reasoned above to interact bidirectionally, potentially adding up to a full gain cycle, and (b) all three components are relevant resources in the COR theory which substantiates gain cycles in the literature. We hypothesize:

Hypothesis 4: Social support, PsyCap and crafting for social job resources are reciprocally interacting and establishing a gain cycle.

## Materials and Methods

### Participants and Procedure

This study is based on three waves of longitudinal data with a time interval of 3 months. Participants from German-speaking countries (Austria, Germany, and Switzerland) were recruited through an online panel data service. Participants received a minimal incentive to participate. Their participation was voluntary, and the anonymity and confidentiality of the data were guaranteed. Participants had to submit their informed consent prior to the study by clicking a check box in the online questionnaire. The composition of the sample was determined to ensure a representative distribution of participant characteristics, that is in line with the studied populations. This was done based on data provided by the census bureau of the respective country (http://www.statistik for Statistik Austria, www.destatis.de for Germany, and www.bfs.admin.ch for Switzerland). Regarding relevant demographic variables (age, organizational tenure, education, and the industrial sectors) it can be stated that the data collected represents the working population of the studied countries very well (for more details see Vogt et al., 2016).

Employees who indicated that they worked more than 20 h per week in an employed job were included in the study. Additionally, age constraints were set to a range of 18–65 years. These criteria resulted in a sample of 1.852 employees who completed the questionnaire in the first wave. After 3 months, 1.229 of the first wave participants participated in the second wave. Six months after the baseline measurement, 995 employees participated in wave three.

Moreover, we tested for the presence of non-random sampling by means of a logistic regression as recommended by Goodman and Blum (1996). Nagelkerke *R*^2^ indicated that the explained variance in all estimated models was not substantial, and therefore no variable systematically contributed to the dropout: PsyCap (*B* = 0.12; *SE* = 0.06; *p* < 0.05; Nagelkerke *R*^2^ = 0.00), social support by managers (*B* = 0.00; *SE* = 0.05; *p* = 0.86; Nagelkerke *R*^2^ = 0.00), social support by colleagues (*B* = 0.12; *SE* = 0.06; *p* < 0.05; Nagelkerke *R*^2^ = 0.00), crafting for social job resources (*B* = 0.12; *SE* = 0.07; *p* = 0.07; Nagelkerke *R*^2^ = 0.00).

Of the final sample across all three waves, 54% were from Germany, 32% were from Austria, and 14% were from Switzerland. There were more male participants (63%) with a mean age of 41.2 years. In the health and social sector worked 10%; 12% in the public/defense/social security sectors; 8% in trading; 9% in the production of goods; 8% in information/communication; 6% in finance/insurance; 7% in technology/science; 5% in education. The remaining participants worked in the real estate, hospitality, transport and building industries. Forty percent had completed an apprenticeship, and 33% had earned a degree from a higher educational institution. The mean organizational tenure was 10.7 years (*SD* = 9.1).

### Measures

All measures that were validated in languages other than German were translated into German and then checked for accuracy, using the back-translation procedure.

*Psychological capital* was measured by the PCQ-12 (Luthans et al., 2007a), which is especially compatible for use in longitudinal research (Avey et al., 2008). Its items apply a 6-point Likert scale ranging from 1 (strongly disagree) to 6 (strongly agree). The “hope” subscale was derived from the State Hope Scale (Snyder et al., 1996). The “optimism” items were adopted from Scheier and Carver’s (1985) measure of optimism. The “self-efficacy” in the workplace items were adopted from Parker (1998), and “resilience” was based on the Resilience Scale (Wagnild and Young, 1993).

*Crafting for social job resources* was assessed by the items of the subdimension *Increasing social job resources* of the Job Crafting Scale (Tims et al., 2012): “I ask my supervisor to coach me”; “I ask whether my supervisor is satisfied with my work”; “I look to my supervisor for inspiration”; “I ask others for feedback on my job performance”; and “I ask colleagues for advice.” All items were rated on a 5-point Likert scale with the endpoints of “never” and “very often.”

We assessed two sources of *social support* that are relevant to a broad range of jobs and organizations. Items are taken from the Management Standards Indicator Tool developed by the UK’s Health and Safety Executive (HSE; Cousins et al., 2004): *Peer support* (“I get the help and support I need from my colleagues”) and *manager support* (“My line manager encourages me”). Both items were selected because they had the highest factor loadings in the two subscales of the HSE indicator tool, that referred to social support (Cousins et al., 2004, p. 129). The items were measured using a 5-point Likert scale ranging from 1 (never) to 5 (very often).

### Data Analysis

The data were analyzed using structural equation modeling techniques with the IBM AMOS 25 software package (Arbuckle and Wothke, 1999). We assessed several nested models using the root mean square error of approximation (RMSEA), the comparative fit index (CFI), the normed fit index (NFI), and the Tucker-Lewis index (TLI) with conventional cut-off values (RMSEA < 0.08; CFI > 0.95; NFI > 0.95; TLI > 0.95) (Schermelleh-Engel et al., 2003). We compared them with the results of chi-square difference tests (Jöreskog and Sörbom, 1993). The error terms of the indicators and latent variables were allowed to covary with the corresponding error terms of the other two waves and according to the resulting modification indices of model tests (Newsom, 2015, p. 126).

## Results

### Cross-Lagged Interactions Between Study Concepts

**FIGURE 1:**
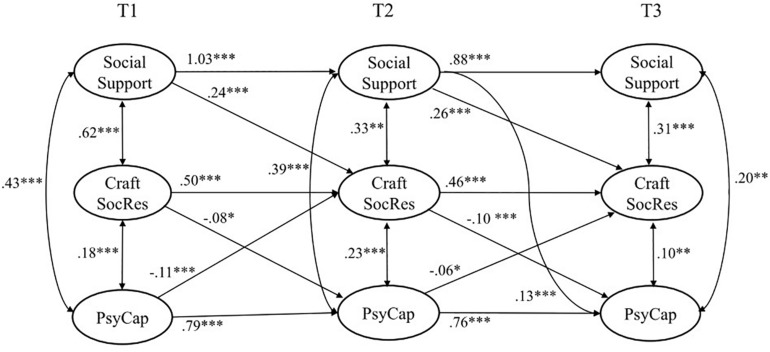
Significant relationships among PsyCap, crafting for social job resources, and social support (*N* = 995). *^∗^p <* 0.05; *^∗∗^p <* 0.01; ^∗∗∗^*p* < 0.001. Tl, wave 1; T2, wave 2; T3, wave 3.

As a first step of the analysis, correlations and reliabilities of scales and respective variables were calculated (Table 1). The fit indices of all tested models are shown in Table 2, which also shows the differences between the hypothesized models and the baseline (i.e., the null model). In meeting the preconditions, all tested models exceeded the null model. To test the hypotheses in the first step of the analysis, a stability model M1 was tested, in which baseline values were predictors of the latent variables. This model was compared to the initial null model and fitted better with the data (M0 vs. M1: Δχ^2^ = 3221.5, Δ*df* = 6, *p* < 0.001). In the second step, a fully mediated causality model M2 (in which PsyCap predicted crafting for social job resources and crafting for social job resources predicted social job resources over all three measurement timepoints) was compared to the stability model M1. The fit of the causality model M2 was not superior to that of the stability model (M1 vs. M2: Δχ^2^ = 3.23, Δ*df* = 6, *ns*). Furthermore, the fully mediated reversed causality model M3 (in which social job resources predicted crafting for social job resources and crafting for social job resources predicted PsyCap) matched the data significantly better than the stability model did (M1 vs. M3: Δχ^2^ = 82.74, Δ*df* = *6, p* < 0.001). Therefore, the cross-lagged paths led to an improved model in comparison to the previously tested models, including temporal stability and simple causality. Finally, we tested the reciprocal model M4 with mediated pathways in both directions (i.e., causal and reversed causal). The statistical quality of this model did not significantly differ from the reversed model (M3 vs. M4: Δχ^2^ = 20.15, Δ*df* = *6, ns*). Notwithstanding, we referred to model M4 for further interpretation because it contained more information regarding the research questions and hypotheses (see Figure 1).

**TABLE 1:** Means (*M*), standard deviations (*SD*), internal consistencies (Cronbach’s alpha), and partial correlations (controlled for age, tenure, and education) of studied variables (*N* = 995).

	***M***	***SD***	**α**	**1**	**2**	**3**	**4**	**5**	**6**	**7**	**8**
1. Support col/Mgmt T1	3.43	0.89	0.52								
2. Support col/Mgmt T2	3.35	0.92	0.55	0.66^∗∗∗^							
3. Support col/Mgmt T3	3.38	0.90	0.58	0.63^∗∗∗^	0.66^∗∗∗^						
4. Job crafting T1	2.61	0.69	0.79	0.50^∗∗∗^	0.34^∗∗∗^	0.43^∗∗∗^					
5. Job crafting T2	2.54	0.69	0.81	0.40^∗∗∗^	0.42^∗∗∗^	0.38^∗∗∗^	0.62^∗∗∗^				
6. Job crafting T3	2.52	0.66	0.79	0.36^∗∗∗^	0.34^∗∗∗^	0.43^∗∗∗^	0.60^∗∗∗^	0.66^∗∗∗^			
7. PsyCap T1	4.50	0.81	0.87	0.36^∗∗∗^	0.31^∗∗∗^	0.32^∗∗∗^	0.23^∗∗∗^	0.11^∗∗^	0.12^∗∗∗^		
8. PsyCap T2	4.47	0.81	0.87	0.28^∗∗∗^	0.30^∗∗∗^	0.30^∗∗∗^	0.11^∗∗∗^	0.16^∗∗∗^	0.12^∗∗∗^	0.75^∗∗∗^	
9. PsyCap T3	4.45	0.79	0.86	0.29^∗∗∗^	0.30^∗∗∗^	0.35^∗∗∗^	0.16^∗∗∗^	0.10^∗∗^	0.15^∗∗∗^	0.71^∗∗∗^	0.74^∗∗∗^

*T1, wave 1; T2, wave 2; T3, wave 3. ^∗∗^*p* < 0.01 and ^∗∗∗^*p* < 0.001.*

**TABLE 2:** Fit statistics of the studied models.

**Model**	**χ*^2^***	**df**	**CFI**	**TLI**	**NFI**	**RMSEA**	**Model comparison**	**Δχ*^2^ -Diff***	***df-Diff***
M0. Measurement model	3927.15	170	0.746	0.686	0.738	0.149			
M1. Stability model	705.64	164	0.963	0.953	0.953	0.058	M0 – M4	3324.4^∗∗∗^	18
M2. Causality model	702.41	158	0.963	0.951	0.953	0.059	M1 – M4	102.89^∗∗∗^	12
M3. Reversed model	622.90	158	0.969	0.958	0.958	0.054	M2 – M4	99.66^∗∗∗^	6
M4. Reciprocal model	602.75	152	0.970	0.958	0.960	0.055	M3 – M4	20.15 ns	6

*^∗∗∗^*p* < 0.001.*

Figure 1 shows that social support at work had no significant cross-lagged effect on PsyCap from T1 to T2 (β = 0.05, *ns*), but it did have an effect from T2 to T3 (β = 0.13, *p* < 0.001). Thus, Hypothesis 1a is partially supported.

The paths from PsyCap to social support at work were not significant at either interval (from T1 to T2: β = −0.06, *ns* and from T2 to T3: β = 0.01, *ns*). Thus, Hypothesis 1b is rejected.

Social support at work had a significant cross-lagged effect on crafting for social job resources from T1 to T2 (β = 0.24, *p* < 0.001) and from T2 to T3 (β = 0.26, *p* < 0.001). Thus, Hypothesis 2a is fully supported. Crafting for social job resources had no significant cross-lagged effect on social support at work from T1 to T2 (β = −0.11, *ns*) or from T2 to T3 (β = 0.01, *ns*). Thus, hypothesis 2b is rejected.

The paths from PsyCap to crafting for social job resources were significantly negative from T1 to T2 (β = −0.11, *p* < 0.001) and from T2 to T3 (β = −0.06, *p* < 0.05). Thus, Hypothesis 3a is not confirmed. Testing for reverse effects, crafting for social job resources had a significant negative cross-lagged effect on PsyCap from T1 to T2 (β = −0.08, *p* < 0.05) and from T2 to T3 (β = −0.10, *p* < 0.001). Thus, hypothesis 3b is rejected.

It was expected that social support, PsyCap, and crafting for social job resources are resources that interact according to the COR theory and constitute gain cycles (Hypothesis 4). Several interactions were found to be substantial, such as the effects of social support on crafting for social job resources. However, a full systematic gain cycle of interacting resources between all stages of measurements was not found.

## Discussion

The aim of the present study was to systematically assess the reciprocal relationships among three important concepts in occupational health psychology: PsyCap, job crafting, and job resources. The latter two were assessed according to their social dimensions, that is, crafting for increasing social job resources and social support from colleagues and supervisors. Even though, these concepts are highly relevant, the research on its reciprocal interactions is limited and is therefore extended by this study. Specifically, we add an overall view of the integrated variables to the research in this area, that goes beyond previous analyses of singular and fragmented associations. An important feature of this study is that it integrates and examines established hypotheses on self-efficacy in the study of PsyCap, crafting and social support. To our best knowledge, this is done here for the first time.

Relationships among the concepts are proposed in the theoretical framework of the JD-R theory, which has influenced the discourse in occupational health psychology and beyond. In this theoretical framework, we integrated two hypotheses: The *enabling hypothesis* (Benight and Bandura, 2004) and the *cultivation of social resources hypothesis* (Schwarzer and Knoll, 2007). Both are directly related to self-efficacy, which is a subdimension of the higher order construct PsyCap.

Gain cycles have been observed, composed of the here studied concepts (e.g., Salanova et al., 2011). We were therefore interested in replicating these findings. To do so, a three-wave full panel design, that can be deemed as representative for the working population of Austria, Germany, and Switzerland, was analyzed with structural equation modeling.

### Social Support at Work Leads to PsyCap

The results indicated that social support at work had a positive effect on PsyCap over time, which was shown by the respective positive longitudinal effect (Hypothesis 1a). This finding supports the *enabling hypothesis* (Benight and Bandura, 2004), which states that social support enables self-efficacy by the positive experiences of success and mastery. For the first time, this hypothesis was expanded to the entire PsyCap complex, here in the context of occupational psychology. This finding is additionally in line with aspect 1 of the JD-R theory, in which it is stated, that job resources amplify personal resources. Social resources exert a positive effect on personal resources, an effect that was not reciprocal (Hypothesis 1b). These results did not support the *cultivation of social resources hypothesis*, which assumes that social resources increase because of higher degrees of self-efficacy.

We assume that the high degrees of self-efficacy and of PsyCap might imply that employees feel more self-sustaining, thus perceiving no need to further increase their social resources.

### Social Support at Work Leads to Crafting for Social Job Resources

Social support at work had a substantial positive effect on crafting for social job resources in all study intervals. Hence, those who had social resources at their disposal showed a high degree of crafting for social job resources and create situations at the workplace according to their needs. Social support at work was therefore shown to be a facilitating factor in job crafting behaviors on the social dimension. Thus, Hypothesis 2a is supported.

It was assumed that crafting for social job resources had the capacity to mobilize social capital and therefore social support at work (Hypothesis 2b), which could not be shown longitudinally. Accordingly, crafting was not successfully resulting in an enhancement of an individual’s social capital (Lin, 2002). Aspect 2 of the JD-R theory, that states that crafting leads to more job resources, cannot be confirmed for the job resource of social support.

It is noticeable, that crafting and social support were already linked at T1 cross sectionally. Potentially reaching a threshold of crafting at this starting point of our study. Afterward, this link remained stable on a high level and did not add any positive effects furthermore over time. This seems reasonable, because the studied concepts were notably constant. The finding leads to the future research question, when und if the effect of crafting can be saturated and individuals consequently do not invest further resources in it?

### Negative Associations Between PsyCap and Crafting for Social Job Resources

PsyCap integrates subdimensions that are highly relevant primers of behavioral outcomes. It was hypothesized that higher PsyCap as a personal resource deploys crafting for social job resources and vice versa. As expected, both components were positively related to one another at the cross-sectional level. Counterintuitively, PsyCap had a negative longitudinal effect on crafting for social job resources over time (between T1 and T2 as well as between T2 and T3: Hypothesis 3a). Once more, this finding discharges the cultivation hypothesis of self-efficacy. Moreover, it supports the notion that the availability of positive personal resources, makes job crafting in the social dimension redundant. This interpretation is also assisted by previous research results, that is (a) that social costs emerge from seeking support in the workplace (Putnam and Mumby, 2014) and (b) that personal resources and respective positive self-concepts lead to more self-reliant strategies in problem solving (Bandura, 2012).

Crafting for increasing social resources did not result in more PsyCap (hypothesis 3b), rather indicated slightly negative results. This finding contributes to the debate on job crafting as an adaptive behavior (Berg et al., 2010). For example, a parent of a young family must meet challenges regarding the compatibility of family and career. He or she adapt to these new situations using crafting behaviors. In the social dimension, crafting then might consume personal resources, which was indicated by the negative effect on PsyCap. That proactive behaviors require resource expenditure is part of an upcoming research stream (Strauss et al., 2017; Parker et al., 2019), to which this finding contributes. In addition, it may be mentioned, that Van den Heuvel et al. (2015) reported, that crafting did influence self-efficacy, but the effect was delayed over the period of a 4-week intervention study. They referred the occurrence of the effect to an additionally, during the studied intervention offered reflection session, that might have helped to establish the effect of crafting on self-efficacy. This underlying systematic may also be relevant for other PsyCap subdimensions. It is reasonable to assume, that the effects of job crafting are not immediately apparent to the person using it. This has so far not been sufficiently researched for PsyCap in organizational contexts. However, we cannot confirm the second aspect of the JD-R theory in this case.

The absence of expected gain cycles might have been caused by this special role of crafting for social job resources as an adaptive strategy in demanding circumstances. To our knowledge, the perspective of crafting as an adaptive strategy has not been studied in the context of gain cycles before.

### Limitations

Some limitations of this study need to be addressed. First, the measures were self-reported, which might have skewed the relationships between the studied concepts by the common method bias (Podsakoff et al., 2012). To counteract this bias, the recommendations of Podsakoff and colleagues were considered (e.g., the participants were informed that there were no wrong or right answers). Additionally, the panel design used in this study reduced the risk of the common method bias (Jakobsen and Jensen, 2015).

Second, the extent to which psychosocial constructs vary or remain stable over time is not yet clear (for a discussion of this phenomenon in the field of personality psychology see Fraley and Roberts, 2005). Nevertheless, the cross-lagged effects presented here indicate a certain stability because the measured constructs were stable over time.

Third, the hypotheses were tested by measuring changes in the levels of PsyCap, crafting behavior, and perceived social support. Even if the present study fulfilled the need for a longitudinal study design, the results did not demonstrate whether the effects would be valid in practice (Taris and Kompier, 2014).

Fourth, although the longitudinal design allowed for measuring the variance in the measured concepts over time, other external influences were not controlled for, which may have influenced the results. A future quasi-experimental design would help to control for external influences (Cook et al., 2002).

### Practical Implications

Crafting is widely promoted as an effective way of designing one’s own working environment. This approach offers employees and organizations options for work design that has been proven in many studies. The results presented here show that crafting is not a stand-alone concept. Rather, the embedding in positive social contexts is relevant. In detail, requesting support can cost colleagues and supervisors time and effort. Consequently, if the absorption of social interaction is costly, this important job resource could remain unused. Therefore, working environments that enable efficient and resource-rich social interaction and support must be ensured. Otherwise, individuals who have resources of the PsyCap dimensions will rely on them and not craft for social support.

## Conclusion

This study advances the knowledge of the interactions among the social resources of social support, crafting for social job resources, and the personal resource of PsyCap, in the realm of occupational health psychology. The study was conducted using a large longitudinal and heterogeneous sample, that included employees working in manifold areas of work and economic sectors. Its results support the enabling hypothesis of social support and yielded mixed support for propositions of the JD-R theory. It additionally contributes to the field, as this research examines basic theories and propositions by means of replication.

Social support at work positively supported PsyCap, in accordance with the enabling hypothesis of self-efficacy. For the first time, this hypothesis was tested in the field of occupational health psychology and extended to the whole PsyCap construct. PsyCap and crafting for social job resources were not supporting each other. We conclude that under the condition of high availability of personal resources, social resources are requested and accumulated less. That is also the case, as crafting might be a behavior that requires substantial resources. Previously observed gain cycles were at that point not replicable. Further research is needed to understand some of these unexpected results regarding demanding and resource depleting side of proactive behaviors at the workplace.

## Data Availability Statement

The datasets generated for this study are available on request to the corresponding author.

## Ethics Statement

Ethical review and approval was not required for the study on human participants in accordance with the local legislation and institutional requirements. The patients/participants provided their written informed consent to participate in this study.

## Author Contributions

The data analysis and manuscript was prepared by PK with support from RB and GB. All authors critically reviewed and contributed to the manuscript, and approved the final version.

## Conflict of Interest

The authors declare that the research was conducted in the absence of any commercial or financial relationships that could be construed as a potential conflict of interest.

## Footnotes

1 Approximately cited 7.500 times in Google Scholar – August 2019.
